# Influence of Epilepsy on the Quality of Life of Patients with Brain Tumors

**DOI:** 10.3390/ijerph18126390

**Published:** 2021-06-12

**Authors:** Stanisław Krajewski, Magdalena Wójcik, Marek Harat, Jacek Furtak

**Affiliations:** 1 Department of Physiotherapy, University of Bydgoszcz, 85-059 Bydgoszcz, Poland; kondycja@gmail.com; 2 Department of Neurosurgery, 10th Military Research Hospital, 85-681 Bydgoszcz, Poland; marek.harat@10wsk.mil.pl (M.H.); jacek.furtak2019@gmail.com (J.F.); 3 Department of Neurosurgery and Neurology, Collegium Medicum in Bydgoszcz, Nicolaus Copernicus University, 87-100 Toruń, Poland; 4 Department of Neurooncology and Radiosurgery, Franciszek Łukaszczyk Oncology Center, 85-796 Bydgoszcz, Poland

**Keywords:** brain tumor, epilepsy, quality of life, driving, memory

## Abstract

Epilepsy is a common consequence of brain tumors, occurring in 35 to 75% of cases. Here we evaluated the influence of epilepsy on the quality of life (QoL) of patients with malignant brain tumors (primary and metastatic) and assessed which areas of function are most affected by epilepsy and brain tumors. Sixty patients undergoing brain tumor surgery at the Neurosurgery Clinic of the 10th Military Research Hospital, Bydgoszcz, Poland (30 with epilepsy and 30 without epilepsy) were studied. Relationships between categorical variables were determined with Pearson’s chi-squared test, while continuous data were analyzed with the Mann-Whitney U-test. A *p* value < 0.05 was considered statistically significant. A multiple regression model was used for multivariate analysis of QoL. Patients with epilepsy more frequently reported memory disorders as a problem in their daily life. There were trends towards greater impairments in social, professional, and family life, sports and recreational activities, and daily physical activities in brain tumor patients with epilepsy rather than those without epilepsy. Higher frequency and generalized seizures significantly and adversely influenced the ability of patients to leave home and drive vehicles, but a proportion of patients with frequent, generalized seizures continued to drive regardless. Patients with generalized seizures considered the adverse effects of taking medicines as significantly disruptive. Memory disorders significantly affect the QoL of patients with epilepsy, and the importance of stopping driving must be emphasized by all healthcare professionals.

## 1. Introduction

Primary brain tumors account for 1–2% of all neoplasms, affecting 5–23 people per 100,000 every year and representing 2–2.5% of all cancer deaths. Although demographic data vary, the annual age-adjusted incidence rate of primary brain tumors in the US is 24.8 for women and 20.3 for men, and the disease is more common in Europe and North America than in South-East Asia and Central and South Africa due to a mixture of environmental (i.e., industrialization) and ethnic and genetic factors. About two-thirds of primary brain tumors are benign, half of which are meningiomas. Of the malignant tumors, about 80% are gliomas, over half of which are highly malignant glioblastomas (GBM) [1,2,3,4].

Data on the incidence of metastases to the brain also vary, but the US National Brain Tumor Society report at least twice as many secondary brain cancer diagnoses than primary brain cancer diagnoses each year, although there are reports of 10-times more secondary than primary diagnoses. Regardless, brain metastases are common, affecting 10–30% of patients with cancer and most commonly arising from primary lung (30–40%), breast (5–19%), skin (melanoma; 7–10%), and colon and rectum (7%) cancers [1,3,5,6,7,8].

Epilepsy affects about 35–70% of patients with a brain tumor and often occurs as an early or first clinical symptom. Seizures most often occur in patients with glioneuronal tumors (70–80%), especially those with fronto-temporal and insular lesions. Epilepsy occurs in 60–70% of patients with gliomas [9,10,11,12,13]. As early as the 1990s, early-onset epilepsy in patients with both high- and low-grade gliomas was found to be a positive predictor of overall survival [14,15,16,17,18]. The type of epileptic seizures and their frequency and prognosis depend on the type, localization, and size of tumor. Seizures are more common in patients with slow-growing, poorly differentiated tumors, but epileptogenesis may also be associated with certain genetic disorders [9,11,12,13].

Epilepsy is rarer in patients with metastatic lesions (15–25%), with melanoma metastases conferring the greatest risk of seizure (11–33% [6]) followed by lung metastases (12.5–20%). In terms of mechanism, metastatic tumors tend to be more circumscribed than infiltrating primary tumors, which may explain the lower frequency of seizures in metastatic patients. Furthermore, metastases cause local microcircuiting disorders resulting in ischemic areas and epilepsy, while slow-growing gliomas result in denervation hypersensitivity as the epileptogenic mode of action [18]. Patients with metastatic tumors of the central nervous system usually quickly deteriorate and die, and the life expectancy is usually less than six months. Patients with primary tumors usually fare slightly better and live a few months longer [8,19].

In one comparison of the quality of life (QoL) of patients with primary tumors and metastases (*n* = 1483), patients with primary tumors had significantly worse cognitive function than those with metastases [19]. By contrast, another study (*n* = 1808) showed that patients with primary tumors seem to have better social and functional wellbeing then those with metastatic brain tumors [20]. In both of these studies, the overall QoL scale results and some QoL subdomains (physical and emotional wellbeing, role functioning, and some symptoms such as fatigue, nausea/vomiting, pain, insomnia, and diarrhea) were not significantly different between groups, perhaps due to significant differences in group sizes and heterogeneity of the study populations [19,20].

Nevertheless, patients with brain tumors can suffer from cognitive dysfunction in their attention, memory, and concentration as well as emotional changes such as anxiety and depression. Furthermore, paresis or paralysis, visual and hearing disturbances, and/or aphasia may also develop secondary to brain tumors. While all these symptoms can deteriorate a patient’s QoL, epileptic seizures are generally regarded as having one of the greatest effects on QoL, even in the absence of a brain tumor [4,9,10,11,12,17,21,22,23]. Patients with brain tumors and epilepsy require multidisciplinary treatment, but the combined use of antiepileptic drugs (AEDs), cancer treatment (chemo- and radiotherapy), and auxiliary therapies exposes patients to drug interactions and adverse effects [11,12,24,25,26,27,28,29,30,31]. Together, the physical consequences of brain tumors, epilepsy, and complex treatment severely impact function in multiple domains including social and family relationships, employment and ability to work, activities of daily living, and all areas of physical activity [10,11,12,32,33,34].

QoL and health-related quality of life (HRQoL) are useful concepts not only for evaluating individual patients but also to guide clinical management to aid recovery. When brain tumors and epilepsy co-exist, it is important to determine the extent to which epilepsy and its associated disorders alter the QoL of a patient with brain cancer [12,22,35,36].

Here we evaluated the influence of epilepsy on the QoL of patients with brain tumors to establish the functional domains most impacted by epilepsy and brain tumors.

## 2. Materials and Methods

### 2.1. Study Design

This study was conducted between June and August 2019 at the Neurosurgery Clinic of the 10th Military Research Hospital, Bydgoszcz, Poland. Sixty patients with supratentorial brain tumors (including metastases) participated: 30 patients with brain tumors and epilepsy, and 30 patients with brain tumors but without epilepsy. The Ethics Committee of the Military Chamber of Physicians (no. 170/19) approved the study protocol.

Participants completed a single self-administered questionnaire in four parts. The first and second parts were administered to all participants to gather demographic details, general information about their cancer, and QoL in the four weeks prior to the survey. Parts three and four were completed only by the study group and gathered information on epilepsy course and QoL influenced by epilepsy in the four weeks prior to the survey. The questionnaire contained both open- and closed-ended questions.

To evaluate the effect of epilepsy on the functioning of patients with brain tumors, study participants were also asked questions regarding their independence, physical activity, professional activity, social activity, and mental state:

Evaluation of independence: Questions were asked regarding: (i) help needed from other people to assist with tasks outside the house (shopping, administrative tasks, and nursery/school runs for children/grandchildren); (ii) disease influence on activities associated with self-care (preparing and eating meals, daily hygiene, and moving around at home); and (iii) limitations in activities associated with physical exercise necessary in daily life (going up or down the stairs, carrying heavy shopping, and carrying out more demanding household chores such as washing the windows and working in a garden). This section could score 4–14 points, where 4 denoted that the disease did not affect the evaluated aspects of the activity and 14 denoted the strongest effect.

Evaluation of physical activity: A total of 3–10 points for questions related to limitations to participating in or giving up sports activities, limitations to daily physical exercises, and becoming less active during leisure time.

Evaluation of professional activity: A total of 2–8 points for questions regarding limitations to professional activity and the quality of performed work, taking into account absence from work, breaks at work, number of working hours, and responsibilities.

Evaluation of social activity: A total of 5–14 points for questions regarding limitations to leaving home, limitations to driving a car, and limitations to social (social meetings) and family life.

Evaluation of mental state: A total of 2–6 points for questions regarding reductions in energy, the will to undertake daily activities, and problems with concentration, planning, and decision-making.

Therefore, the overall score ranged from 16 to 52 points. A score of 16 was considered an individual baseline for subjects in the pre-disease period, i.e., a score of 16 at the time of completing the questionnaire meant that the disease did not have any impact on the assessed aspects of the respondent’s life. A score of 52 points was possible by selecting the lowest activity for all spheres of response assessed.

### 2.2. Statistical Analysis

To evaluate the influence of the disease(s) on QoL, the percentage reduction in analyzed aspects of QoL and in overall QoL, was calculated. Data were analyzed using Statistica v13.1 (StatSoft, Hamburg, Germany). Continuous data are presented as descriptive statistics, including mean and standard deviation (SD), while categorical data are presented as numbers and percentages. Relationships between categorical variables were determined with Pearson’s chi-squared test, while continuous data were analyzed with the Mann–Whitney U-test. A *p* value < 0.05 was considered statistically significant. A multiple regression model was used for multivariate analysis of QoL.

## 3. Results

The demographic and clinical data of the study respondents are shown in Table 1. Twelve women and 18 men (aged 47.2 ± 13.5 [23–75 years]) had brain tumors and epilepsy, while 21 women and nine men (age 46.7 ± 15.4 [25–72 years]) had brain tumors alone. There was a significant difference in the number of men and women between the study and control groups (*p* = 0.020). The time from diagnosis of the brain tumor was over one year in 21 (35.0%) patients, from six months to one year in 11 (18.3%) patients, and less than six months in 28 (46.7%) patients; the time from diagnosis did not differ between groups (*p* = 0.341).

Fifty patients (83.3%) had primary brain cancer (86.7% in the study group and 80.0% in the control group) and 10 (16.7%) had metastases to the brain (13.3% in the study group and 20% in the control group; not significantly different between study and control groups, *p* = 0.488) All patients underwent surgery. In 28 (46.7%) patients, the procedure was preceded by a biopsy. Combined radio- and chemotherapy was given to 12 (20.0%) patients, while five (8.3%) patients underwent radiotherapy alone.

With regard to specific symptoms other than seizures, only the frequency of memory disorders was different between groups, with memory disorders more common in brain tumor patients with epilepsy (*p* = 0.044; Table 2).

Overall QoL was compared based on gender, duration of disease, tumor type, and radio- and chemotherapy use. Although noting that there was only a small number of participants in each subgroup, the level of statistical significance between them has been waived. There were no statistically significant differences between groups for any of these variables (Table 3).

A multiple regression model significantly predicted QoL (F = 4.39, *p* < 0.044), with an R^2^ of 0.131. When corrected for age and treatment, gender was a significant predictor of QoL (β = 0.362, *p* = 0.045). Age, sex, and treatment were not independent predictors of QoL (Appendix A, Table A1). There were no statistically significant differences in QoL parameters nor overall QoL score between men and women (Appendix A, Table A2).

All brain tumor patients, regardless of epilepsy status, experienced deteriorated function in all QoL domains after diagnosis. The smallest deteriorations were in professional (43.3% in the study group, 33.3% in the control group) and social (44.4% and 37.7%, respectively) activities, while largest deteriorations were for physical activity (65.7% and 64.3%) and mental state (62.5% and 52.5%). There were no significant differences in any QoL domain between groups, although higher values (worse status) were noted in the study group for four out of the five studied domains (Table 4).

To examine the impact of seizure severity on QoL, the study group (the 30 patients with epilepsy) was divided into patients with frequent seizures (several seizures a month; *n* = 13, 43.3%) and those with rare seizures (seizures occurring several times a year; *n* = 17, 56.7%). Subjects experiencing more frequent seizures less left the home less often (*p* = 0.034) and were less tolerant of antiepileptic drugs (*p* = 0.044) than those with less frequent seizures. There was no significant difference between the study and the control groups with respect to the frequency of leaving home. With respect to changes to driving a car, the study and the control groups did not differ, while patients with frequent seizures were significantly more likely to stop driving a car than those with rare epileptic seizures (*p* = 0.038; Table 5).

Among patients with epilepsy, 11 (36.7%) had generalized seizures and 19 (63.3%) patients had focal seizures. Patients with generalized seizures stopped driving vehicles significantly more often than those with focal seizures (*p* = 0.044; Table 5), but over half (53.4%) of patients with epilepsy did not stop driving, which included some patients with generalized seizures (Table 5 and Table 6).

## 4. Discussion

In our experience, epilepsy is the most frequently observed symptom in patients with brain tumors, and it is often the first symptom (Table 2). This is consistent with epidemiological reports on brain tumors and epilepsy, which are reported to co-exist in 30–75% of cases [10,37,38,39] or even up to 90–100% in some reports [9]. Epilepsy as the first symptom of a brain tumor is reported to occur in 10–50% of cases [37,38,39]. According to Maschio et al., epileptic seizures are an initial symptom of brain tumors in 20–40% of patients, while 20–45% of patients develop epilepsy as the tumor progresses [12].

While QoL was universally affected by having a brain tumor, epilepsy did not further deteriorate the studied QoL domains. However, a patient’s QoL also includes his or her mood, including complex facets of cognition and emotion. Even mild cognitive deficits may be of functional and psychosocial consequence to patients with brain tumors [31,40], and cognitive disorders are a frequent cause of complications associated with co-existent brain tumors and epilepsy [41]. Brain tumors alone pose a direct threat to cognitive function [28,40], with memory and attention disorders observed in over 60% of patients with brain tumors [42]. Epilepsy and epileptic drugs are also reported to contribute to cognitive disorders, with memory and attention the most frequently reported side-effects of AEDs [43]. Aldenkamp et al. reported that the frequency of memory disorders in people with epilepsy was between 20% and 50%, and some patients consider the cognitive consequences of epilepsy to be more exhausting than seizures [44].

The severity of cognitive impairment (including memory problems) in patients with brain tumors and concomitant epilepsy depends on many factors, including tumor size, location, histology, growth rate, patient age, cardiovascular risk, and germline and tumor genetic factors. In addition to local damage, brain tumors also cause global cognitive dysfunction by disrupting cognitive networks. Attention, memory, and executive function are most frequently affected [28,45,46]. Cognitive reserve (CR) also affects functioning, with individuals with high levels of CR able to maintain daily functions despite the accumulation of significant neuropathology. As a result, patients with higher CR levels experience less of an effect from primary brain tumor-specific symptoms on QoL [47].

Surgical treatment, radiotherapy, or chemotherapy reduce tumor load and improve cognitive functioning but can also cause cognitive deficits [6,48]. For example, surgery most affects memory and executive function [49]. Radiotherapy can lead to a significant but usually transient cognitive disability in 50–90% of patients. Complications of radiotherapy include radiation necrosis and encephalopathy, which can result in dementia [50]. Learning, memory, information processing speed, and executive function disorders associated with chemotherapy have been described as “brain chemo” or “cancer-related cognitive impairment” [51,52,53].

Memory-related complaints are most common in patients with temporal epilepsy and can be divided into three types: (i) transient epileptic amnesia (recurrent episodes of amnesia); (ii) accelerated long-term forgetting (newly acquired memories fade over days to weeks); and (iii) remote memory impairment (loss of memories for personal or public facts or events from the distant past) [54]. Here, memory disorders were reported in 3.3% of patients with brain tumors alone but in 20% of patients with co-existent epilepsy. Both epilepsy and AEDs can affect cognitive function and behavior [25,28] and, in patients with brain tumors and epilepsy, the probability of delayed adverse effects of AEDs, such as cognitive disorders or depression, is higher than in patients with epilepsy not associated with a tumor. Although AEDs may not be the sole cause of these adverse effects, they may be a contributing factor [29]. According to the International Bureau for Epilepsy, over half of adult patients reported cognitive disorders caused by AEDs that affected work, leisure activities, and their family and social life [27].

Brain cancer affects the entire life of an affected patient. According to some studies, men are more prone to stress and to lowering QoL [55]. In another study, women tend to report worse QoL and more distress compared to males [56]. On the other hand, studies conducted on 236 patients operated on because of a brain tumor in Bydgoszcz, Poland did not show a statistically significant difference between gender and QoL [57]. Here we found gender as a significant predictor of QoL in multivariate analysis, but comparing mean values in the men’s and women’s groups, we found no differences.

Many patients are forced to give up their social roles [32,58], as do patients with epilepsy. The visible symptoms and consequences of treatment of brain tumors, such as paresis, speech disorders, and deteriorated appearance, together with a label of being “epileptic”, may lead to frustration and adversely impact social and/or interpersonal relationships [12,32]. Indeed, in one study, stigmatization was reported to be the most difficult consequence of epilepsy [33]. Here, over half of patients with brain tumors and epilepsy limited their social activities and half of these patients limited their leaving home.

Patients with brain tumors and epilepsy are also particularly vulnerable to changes in their employment. The majority of people diagnosed with brain tumors stop working [59] and, similarly, epilepsy is a known barrier to starting and retaining a job [60]; indeed, nearly a quarter of both groups of patients studied here discontinued work. Although our patients reported that both epilepsy and brain tumors limited their professional functioning, their coexistence was not additive.

The severity of epilepsy appeared to influence its impact on the QoL of patients with brain tumors. Frequent epileptic seizures were reported in 43.3% of our patients and generalized seizures in 36.7% of subjects. Stavem et al. reported that patients not experiencing any epileptic seizures for six months had better results for all QoL parameters evaluated using the QOLIE-31 questionnaire compared to patients with one seizure a week or with daily seizures [61]. In patients hospitalized for epilepsy, generalized seizures contributed to a deterioration in QoL to a comparable extent to very frequent epileptic seizures and drug resistance [62]. When considering QoL in patients with epilepsy, limitations to driving are frequently listed as an adverse consequence of the disease [63]. Despite treatment, 30% to 40% of patients with epilepsy do not have full seizure control, so patients with drug-resistant epilepsy and frequent seizures, particularly generalized ones, are at increased risk of a car accident [26]. Rosińczuk-Tonderys et al. reported that 29% of driving patients with epilepsy felt that the disease made driving a car impossible but they still used a car, while 57% reported that their condition did not interfere with driving [64]. We found that 30.8% of participants of our study with frequent seizures and 27.3% of patients with generalized seizures did not stop driving. In light of these results and the published literature, this result is concerning. In Poland, driving is regulated by the Regulations of the Minister of Health, 2019. When epilepsy is diagnosed, the neurologist informs the patient that he/she is not allowed to drive, and the patient signs the medical record to confirm that they have received and understand that information. A person with epilepsy may drive again after a six-month seizure-free period after the first seizure if he/she does not require AEDs or after two years (10 years for professional drivers) if AEDs are required. The neurologist does not inform the authorities about the disease, so the responsibility to adhere to the rules lies with the patient. Although driving vehicles with epilepsy is not a crime in Poland, there may be criminal sanctions if, as a result of an illness of which the person is aware, it leads to a road accident. The problem of driving with epilepsy and brain tumors is also exacerbated by the organic brain disease, which can impair decision-making by the patient. Therefore, during management, healthcare professionals should emphasize not only the importance of stopping driving but also the important role played by the family or caregivers in controlling the patient’s activities. In this respect, one solution could be to obligate doctors to notify the relevant authorities to withdraw the driving license in these cases. Regardless of whether the patient suffers frequent or infrequent generalized or focal seizures, they should not drive a car both due to the side-effects of AEDs and the risk associated with experiencing a seizure when driving.

Here, 46.2% of patients significantly reduced their frequency leaving home due to frequent epileptic seizures, while 23.1% of subjects left home less often for this reason. Kobau et al. found that adults who had recently had a seizure reported significantly more days of limited activity compared to people with active epilepsy who had not suffered a recent seizure [35]. Eighty-five per cent of the studied patients with frequent epileptic seizures suffered from adverse effects from their drugs, and 23.1% of patients found that they could not cope with these adverse events. The QoL of patients with brain tumors and epilepsy represents the combined effects of the complex management of two diseases. Epilepsy accompanying brain tumors is frequently drug-resistant, complicating AED therapy and incurring a burden on the patient [12,65]. Adverse events from AEDs are reported to be more frequent in patients with epilepsy associated with brain tumors than in the population suffering from epilepsy alone [9]. Clinically, the adverse events from AEDs may be the most important factor determining QoL second to seizure frequency [30].

The presence of epilepsy is considered to be the most important risk factor for long-term disability in brain tumor patients [11,12,23], impacting both mental and physical fitness and thus independence. Nearly all of our patients limited their daily physical activity due to epileptic seizures, the brain tumor, or their coexistence. Patient with epilepsy and suffering from recurring seizures are usually aware of their limitations, leading to anxiety and withdrawal from many physical activities [32,66]. Brain tumor patients similarly reduce their physical activity, and this negatively affects their QoL [67]. Nearly one-third of our patients with brain tumors and epilepsy played sports less often or less intensively than previously, while one third gave up sports altogether, resulting in a 65.7% and 64.3% reduction in physical activity in patients with and without epilepsy, respectively.

Patients with neurological deficits after brain tumor surgery are provided rehabilitation as soon as possible after the procedure [68,69,70]. However, given our results, rehabilitation may also be useful in people not manifesting neurological deficits, given the reported decline in physical activity due to cancer and epilepsy. While the objective effect of physiotherapy on many disease symptoms is small [71], mobilization through movement and exercise, in combination with psychological therapy, may positively influence areas of physical activity that are not permanently affected [72,73,74].

This study has several limitations. First, there was no assessment of QoL before disease onset, which may have resulted in overestimates of the impact of the disease on the assessed parameters. Second, we did not specifically analyze the tumor type, size, and location or detail the specific AEDs used by participants, and further research is necessary to establish the impact of these parameters on QoL in co-existent disease. Finally, while the study group of 60 patients is relatively small and the compared groups were heterogeneous in terms of gender and some of the first symptoms of the disease, but these parameters did not seem to have a major impact on the QoL parameters studied here.

## 5. Conclusions

Brain tumor patients with epilepsy were more likely to report memory disturbances than patients without epilepsy. While the differences between groups were not significant, both brain tumors and epilepsy resulted in deteriorations in QoL parameters, especially mental wellbeing and physical activities. Of concern, over half of patients with epilepsy and brain tumors, including some with frequent and generalized seizures, did not stop driving, and the serious risks and consequences of continuing to drive by people with epilepsy must be clearly emphasized by healthcare professionals.

## Figures and Tables

**Table 1:** Demographic and clinical data of the study participants.

		Study Group		Control Group		Total		*p* Value *
		n	%	n	%	n	%	
Gender	Female	12	40.0	21	70.0	33	55.0	0.020
	Male	18	60.0	9	30.0	27	45.0	
Time from diagnosis	Over 1 year	13	43.3	8	26.7	21	35.0	
	6 months to 1 year	4	13.3	7	23.3	11	18.3	0.341
	Less than 6 months	13	43.3	15	50.0	28	46.7	
Type of cancer	Primary	26	86.7	24	80.0	50	83.3	0.488
	Metastasis	4	13.3	6	20.0	10	16.7	
Treatment	Chemotherapy	0	0.0	0	0.0	0	0.0	1.000
	Radiotherapy	5	16.7	0	0.0	5	8.3	0.020
	Both chemo- and radiotherapy	9	30.0	3	10.0	12	20.0	0.052
	Symptomatic treatment	6	20.0	8	26.7	14	23.3	0.541
Tumor biopsy		14	46.7	14	46.7	28	46.7	1.0
Other chronic diseases		18	60.0	16	53.3	34	56.7	0.602
Age		M; SD [range]		M; SD [range]		M; SD [range]		
		47.2 ± 13.5 [23–75]		46.7 ± 15.44 [25–72]		47.0 ± 14.37 [23–75]		0.790

* *p* value—difference between study and control group.

**Table 2:** First symptoms, frequency, and type of seizures.

Symptom		Study Group	Control Group	Total	*p* Value *
		n (%)	n (%)	n (%)	
Headaches		3 (10.0%)	6 (20.0%)	9 (15.0%)	0.278
Dizziness		3 (10.0%)	6 (20.0%)	9 (15.0%)	0.278
Consciousness disturbances		1 (3.3%)	0 (0%)	1 (1.7%)	0.313
Nausea, vomiting		0 (0%)	1 (3.3%)	1 (1.7%)	0.313
Fainting, loss of consciousness		0 (0%)	2 (6.7%)	2 (3.3%)	0.150
Most of the above		1 (3.3%)	6 (20.0%)	7 (11.7%)	0.044
Coordination disorders		2 (6.7%)	1 (3.3%)	3 (5.0%)	0.553
Balance disorders		2 (6.7%)	3 10.0%)	5 (8.3%)	0.640
Somnolence		1 (3.3%)	2 (6.7%)	3 (5.0%)	0.553
Memory disorders		6 (20.0%)	1 (3.3%)	7 (11.7%)	0.044
Aphasia		4 (13.3%)	4 (13.3%)	8 (13.3%)	1.000
Face tingling		0 (0%)	2 (6.7%)	2 (3.3%)	0.150
Muscle weakness		2 (6.7%)	2 (6.7%)	4 (6.7%)	1.000
Gait disturbance		0 (0%)	1 (3.3%)	1 (1.7%)	0.313
Blurred vision		0 (0%)	2 (6.7%)	2 (3.3%)	0.150
Foreign smells		0 (0%)	1 (3.3%)	1 (1.7%)	0.313
Bradykinesia		1 (3.3%)	0 (0%)	1 (1.7%)	0.313
Behavior changes		1 (3.3%)	0 (0%)	1 (1.7%)	0.313
No symptoms, tumor found by chance		0 (0%)	5 (16.7%)	5 (8.3%)	0.020
Epilepsy		30 (100%)	-	-	
Epilepsy as the first symptom		15 (50%)	-	-	
Seizure frequency	Few times per week	0 (0%)	-	-	
	Few times per month	13 (43.3%)	-	-	
	Few times per year	17 (56.7%)	-	-	
Seizure type	Generalized	11 (36.7%)	-	-	
	Focal	19 (63.3%)	-	-	

* *p* value—difference between study and control group.

**Table 3:** Overall quality of life (QoL) scores in patients with brain tumors with and without epilepsy.

		Study Group	Control Group	Total
		Mean, SD (*n*)	Mean, SD (*n*)	Mean, SD (*n*)
Gender	Female	33.6 ± 6.72 (12)	32.6 ± 7.95 (21)	33.0 ± 7.44 (33)
	Male	34.8 ± 6.90 (18)	33.0 ± 9.96 (9)	34.2 ± 7.9 (27)
				*p* = 0.530
Time from diagnosis	Over 1 year	35.2 ± 6.43 (13)	33.4 ± 9.02 (8)	34.5 ± 7.35 (21)
	6 months to 1 year	34.8 ± 4.03 (4)	36.6 ± 7.72 (7)	35.9 ± 6.44 (11)
	Less than 6 months	35.2 ± 6.42 (13)	30.6 ± 8.27 (15)	31.9 ± 8.08 (28)
				*p* = 0.173
Type of cancer	Primary	33.5 ± 6.35 (26)	32.1 ± 9.21 (24)	32.9 ± 7.80 (50)
	Metastasis	39.5 ± 7.85 (4)	35.2 ± 3.60 (6)	36.9 ± 5.72 (10)
				*p* = 0.126
Treatment	Chemo- and radiotherapy	35.4 ± 7.56 (14)	41.0 ± 4.36 (3)	36.4 ± 7.33 (17)
	No chemo- and radiotherapy	33.4 ± 6.03 (16)	31.8 ± 8.31 (27)	32.4 ± 7.51 (43)
				*p* = 0.386

**Table 4:** Subgroup and overall QoL scores in patients with brain tumors with and without epilepsy.

Parameter	Group	Mean, SD	Reduction in Parameter *	*p* Value **
Independence	Study	8.7 ± 2.01	47.0%	0.817
	Control	8.8 ± 2.49	48.0%	
Physical activity	Study	7.6 ± 1.94	65.7%	0.970
	Control	7.5 ± 2.24	64.3%	
Professional activity	Study	4.6 ± 2.64	43.3%	0.448
	Control	4.0 ± 2.80	33.3%	
Social activity	Study	9.0 ± 2.01	44.4%	0.367
	Control	8.4 ± 2.58	37.7%	
Mental state	Study	4.5 ± 1.28	62.5%	0.275
	Control	4.1 ± 1.41	52.5%	
Overall assessment	Study	34.3 ± 6.74	50.8%	0.579
	Control	32.7 ± 8.43	46.4%	

* Note: Percentage changes (reductions in a given parameter) were calculated as follows: the minimum value (no effect) was subtracted from the maximum value of the indicator showing the strongest effect of the disease, e.g., for the overall score: 52 − 16 = 36, where 36 is 100%. The value of the parameter calculated for the study group was 34.3 so, compared to the pre-disease condition (16 points), it decreased by (34.3 − 16 = 18.3), or 50.8%. ** *p* value—difference between study and control group.

**Table 5:** Influence of epileptic seizure frequency on selected aspects of functioning in patients with brain tumors.

		Frequent Seizures *n* = 13	Rare Seizures *n* = 17	Total *n* = 30	*p* Value *
		*n* (%)	*n* (%)	*n* (%)	
Frequency of leaving home	Not influenced by epilepsy	4 (30.8)	8 (47.1)	12 (40.0)	
	Moderate influence	3 (23.1)	8 (47.1)	11 (36.7)	*p* = 0.034
	Significant limitation	6 (46.2)	1 (5.9)	7 (23.3)	
Experienced adverse effects of antiepileptic drugs	Not experienced	2 (15.4)	10 (58.8)	12 (40.0)	
	Experienced but coped with	8 (61.5)	4 (23.5)	12 (40.0)	*p* = 0.044
	Experienced and unable to cope with	3 (23.1)	3 (17.7)	6 (20.0)	
Driving a car	Did not stop	4 (30.8)	7 (41.2)	11 (36.7)	
	Drives less frequently	0	5 (29.4)	5 (16.7)	*p* = 0.038
	Ceased completely	9 (69.2)	5 (29.4)	14 (46.6)	

* *p* value—difference between study and control group.

**Table 6:** Influence of type of epileptic seizure on driving vehicles in patients with brain tumors.

Driving a Car	Generalized Seizures *n* = 11	Focal Seizures *n* = 19	Total *n* = 30	*p* Value *
	*n* (%)	*n* (%)	*n* (%)	
Did not stop	2 (18.2)	9 (47.4)	11 (36.7)	
Drives less frequently	1 (9.1)	4 (21.1)	5 (16.7)	*p* = 0.044
Ceased completely	8 (72.7)	6 (31.6)	14 (46.6)	

* *p* value—difference between study and control group.

## Data Availability

All data relevant to this study are reported within the manuscript.

## Appendix A

**Table A1:** Multivariate analysis of quality of life (QoL).

Dependent Variable	Independent Variables	BETA (β)	SE	t	*p* Value
Step 0					
QoL R = 0.39; R^2^ = 0.151 F(3,27) = 1.615; *p* = 0.208	Age	−0.052	0.127	−0.255	0.800
	Gender	0.350	3.136	1.695	0.102
	Treatment	−0.126	2.002	−0.652	0.520
Step 1					
QoL R = 0.387; R^2^ = 0.150 F(2,28) = 2.473; *p* = 0.102	Gender	0.326	0.181	1.806	0.082
	Treatment	−0.141	0.181	−0.779	0.442
Step 2					
QoL R = 0.362; R^2^ = 0.101 F(1,29) = 4.398; *p* < 0.044	Gender	0.363	0.173	2.097	0.045

BETA (β), standardized beta coefficient; t values, Bł; SE, standard error.

**Table A2:** Subgroup and overall QoL scores in females and males with brain tumors with and without epilepsy.

Parameter	Group	Female Mean, SD	Male Mean, SD	*p* Value
Independence	Study	8.5 ± 2.02	8.8 ± 2.05	0.849
	Control	9.0 ± 2.64	8.6 ± 2.24	0.680
Physical activity	Study	7.8 ± 2.09	7.6 ± 1.89	0.176
	Control	7.7 ± 2.67	7.1 ± 2.62	0.934
Professional activity	Study	3.8 ± 2.45	5.1 ± 2.68	0.253
	Control	3.6 ± 2.67	4.8 ± 3.07	0.592
Social activity	Study	8.8 ± 2.01	9.1 ± 2.05	0.751
	Control	8.4 ± 2.67	8.3 ± 2.50	0.773
Mental state	Study	4.8 ± 1.11	4.3 ± 1.36	0.751
	Control	4.0 ± 1.41	4.2 ± 1.48	0.967
Overall assessment	Study	33.6 ± 6.72	34.8 ± 6.90	0.641
	Control	32.6 ± 7.95	33.0 ± 9.96	0.741
